# Perceived Needs of People with Intellectual Disabilities Undergoing the Ageing Process

**DOI:** 10.3390/healthcare13212728

**Published:** 2025-10-28

**Authors:** Elena Felipe-Castaño, Matilde Vivas, Ana Isabel González-Contreras, Raquel Braga-Santos

**Affiliations:** 1 Departament of Psychology, University of Extremadura, 10003 Cáceres, Spain; anaisabelgc@unex.es; 2 Plena Inclusión Extremadura, Avd. Juan Carlos I, 47, Mérida, 06800 Badajoz, Spain; matilde.vivas@plenainclusionextremadura.org; 3 Asociación Juan XXIII, 36160 Pontevedra, Spain; residencia@asociacionjuanxxiii.org

**Keywords:** intellectual disability, ageing, perceived needs, solutions

## Abstract

Background/Objectives: Ageing in people with intellectual disabilities is a social reality that poses challenges for the individual, their family and social environment, and the direct care services. The main objective of this study was to identify the perceived needs of a sample of people with intellectual disabilities currently undergoing the process of ageing. Methods: Cluster random sampling was used to select the 211 individuals with intellectual disabilities who took part in the study. The participants were aged between 42 and 75 years (*M* = 56.18; *SD* = 8.60; *Mdn* = 55), of whom 55.9% (*n* = 118) were women and 43.6% (*n* = 92) were men. Part of the sample completed the Interview for the Needs Evaluation of Ageing People with Disabilities interview by themselves, while the rest did so with support. Results: All the participants identified needs across all the areas assessed, and differences were found according to sex (δ = 0.24–δ = 0.26), age group (δ = 0.20), living arrangements (δ = 0.16–δ = 0.26) and severity of intellectual disability (ε^2^ = 0.03–ε^2^ = 0.08). No differences were found between those who completed the interview independently and those who did so with assistance. Conclusions: The perceived needs of people with intellectual disabilities undergoing the ageing process are complex and multidimensional. Understanding these needs could potentially be useful when designing person-centred needs-based programmes and service models.

## 1. Introduction

In recent decades, the life expectancy of people with intellectual disabilities (IDs) has increased significantly in developed countries, thanks to advances in healthcare, social services and technology [1,2]. Examples include improved living conditions, the consolidation of person-centred care models, more equitable access to healthcare services and the development of assistive technologies [3].

According to data from the Survey on Disability, Personal Autonomy and Dependency Situations [4], around 54.1% of the total estimated population with ID in Spain is aged over 40, indicating that this social group is undergoing a significant ageing process.

Although ageing is a universal and natural process, it can present distinct characteristics in people with ID which are shaped by specific life, social and personal factors. For example, the physiological decline in people with ID tends to occur earlier. The onset of this premature aging process is estimated by some researchers to be around the age of 45 [5]. From that age onwards, increased rates of cognitive decline, physical and chronic illnesses, social isolation and barriers to accessing adapted services have all been observed [6,7].

However, despite its importance, the scientific literature continues to highlight our lack of knowledge and understanding of the life trajectories of people with ID. The ageing process of people with IDs presents significant challenges not only for the individual but also for their families and care models. These include difficulties accessing mainstream healthcare services, a lack of adequately trained staff, rigid funding structures, the unsuitability of community housing for complex needs and the absence of advance planning for end-of-life care [8].

In addition, current health and social care systems are not designed to respond effectively to the changing needs of this population as they age [9]. Another critical challenge is the question of shared dependency between people with ID and their older carers, which is further exacerbated by a lack of planning and appropriate support resources [10]. Other factors such as bereavement, changes in routine, institutionalisation or the loss of key support figures can also have a significant impact on the emotional wellbeing of these individuals [11].

People with ID therefore present a differentiated health needs profile [12]. In addition, according to the findings of research and support organisations, as they age, new needs emerge which impact both the individuals and their families [13]. From a broad perspective, these needs are found across critical areas such as physical and mental health—including signs of dementia in up to 44% of cases, functional decline, sensory impairment, and self-determination, among others [14].

In fact, it is the level of disability rather than the chronological age of the person that determines the degree and nature of these needs [15]. The research also highlights differences in perceived needs depending on the type of ID, health status, and social and family circumstances [16,17,18]. Physical well-being—which encompasses physical health, mobility, personal safety, injuries or harm, and other health-related needs [19]—is the most common need identified by direct care professionals. It is also important to note that needs can evolve depending on the nature of the ID itself or on other broader social developments. For this reason, they need to be studied over time and from multiple disciplinary perspectives [20].

Despite the valuable body of evidence available in the literature, certain gaps persist regarding how these needs are perceived by the individuals with ID. Much of the literature is based on assessments carried out by professionals and support staff—not by the people with the ID themselves—which may have prevented emerging or subjective needs from being detected or reported [21]. Similarly, some studies adopt a fragmented approach that focuses on specific areas such as physical health, functionality or residential environment, without offering an integrated perspective that connects the multiple dimensions of well-being and quality of life in this population.

For all these reasons, the main objective of the study was to describe the perceived needs of a sample of aging individuals with ID and to analyse differences according to sex, age group, severity of the ID and usual residence (including cohabitation arrangements).

Understanding the perceived needs of individuals with ID will help to identify priorities, enable advance planning of resources, and support active, healthy ageing that fosters autonomy, emotional well-being and social participation.

## 2. Materials and Methods

### 2.1. Participants

The study involved 211 individuals with ID, aged between 42 and 75 years, with a mean age of 56.18 years (*SD* = 8.60; *Mdn* = 55). All the participants came from two of Spain’s Autonomous Communities and were already receiving support from centres and services aimed at people with IDD and/or from services aimed at older adults. Of the total sample, 118 (55.9%) were women and 92 (43.6%) were men. The mean age for women was 56.3 years (*SD* = 7.92), and for men 55.93 years (*SD* = 9.43).

Participants were selected through non probabilistic cluster sampling, using the care services (occupational or day centre) as the sampling unit. Sixteen services from two Spanish Autonomous Communities were selected. The following inclusion criteria were used for selecting the participants from each centre: (a) they were aged between 40 and 75 years; (b) presented no cognitive decline; and (c) provided informed consent either themselves or through their families and legal guardian to participate in the study. Criteria for exclusion were (a) inability to complete to the interview, either by themselves or with assistance, or (b) not meeting the inclusion criteria. In cases where participants completed the interview with assistance, the support was provided by direct care staff who had known the person with IDD for a minimum of six months prior to the interview.

Additional sociodemographic characteristics of the participants are presented in Table 1. Two groups were created based on the age of the participants: an ‘early onset aging’ group of people aged between 40 and 55 who already evidenced changes in health, functional autonomy and support needs, and a second ‘advanced aging’ group of individuals aged between 56 and 75 among whom the risk of chronicity, multimorbidity and social vulnerability could worsen [18,22].

No statistically significant differences were found in the mean age by sex, nor in the distribution of participants by sex across the described sociodemographic variables. However, significant differences were observed in relation to their usual place of residence, with more men living with family members.

### 2.2. Instruments

Sociodemographic Data. Information was collected on the participants’ age, sex, usual place of residence and cohabitation arrangements, level of ID (based on service records), and officially recognised level of dependency (based on service records according to Law 39/2006 of the Spanish Ministry of Health and Social Affairs), as well as about the person who completed the ENDE interview.

The Interview for the Needs Evaluation of Ageing People with Disabilities (ENDE, [from the Spanish abbreviation of Entrevista para la Evaluación de Necesidades de personas mayores con Discapacidad] [23]) is based on the quality of life model proposed by Schalock and Verdugo [24,25] which views these individuals’ needs as a fundamental principle [26,27]. The interview comprises 93 items and assesses four main areas: (a) Perceived needs (subdivided into six categories: personal health, financial resources, health care resources, social services resources, physical and social barriers, and support measures and networks); (b) Personal problems; (c) Future outlook; and (d) Solutions. The response options are non-exclusive, allowing respondents to select as many as they consider applicable. As a result, the response percentages can be interpreted independently of the total number of responses.

Two versions of the interview exist: one for individuals with ID, and another for direct support professionals working with people with ID. The version designed for individuals with ID can be completed by the person with the disability either with or without the help of another person or by the support figure on behalf of the person with the disability. The ENDE included appropriate validity indicators [23].

### 2.3. Procedure

A non-experimental, cross-sectional, and quantitative design was used. The study was part of a broader investigation on cognitive functions in individuals with IDD undergoing the aging process.

First, an application for approval for the study and for the informed consent procedure was sent to the Bioethics Committee of the Association Juan XXIII (approval date 12 November 2019). The study adhered to the international ethical standards established by the APA and complied with both the principles of the Declaration of Helsinki and current data protection legislation (Organic Law 3/2018 dated 5 December). Recommendations regarding research involving individuals with ID were also taken into account [28].

A complete list of all the care centres for individuals with ID was drawn up, from which sixteen centres were selected (eight from each Community) using computer-generated random numbers. These centres were subsequently contacted and invited to take part in the study. All of the centres agreed to participate. The participants were selected purposively by the management based on the proposed inclusion/exclusion criteria. The next step involved visiting the selected centres to explain the aim of the study, the procedure for selecting the participants, the process for obtaining informed consent, and the administration and completion of the ENDE interview. Informed consent was obtained prior to the interview, with support provided for participants with greater difficulties and, where applicable, additional consent sought from a family member or legal guardian. Guidance was provided regarding the specific inclusion and exclusion criteria involved. The centres were offered two options: the individual with ID could either complete the interview by themself (self-application) or with the support of another person. A total of 50.2% (*n* = 106) of the participants completed the interview by themselves, while 49.8% (*n* = 105) did so with the assistance of another person. In this case, the support persons were direct care staff who had known the individual with ID for at least six months prior to the interview.

Once the interview had been completed, the data were coded in an SPSS file, with identifying information removed and replaced by numerical codes to ensure anonymity. The data file was held by the lead researcher for subsequent analysis.

### 2.4. Data Analysis

The data were coded and analysed using the statistical software package SPSS 25.0. (IBM SPSS Statistics, Chicago, IL, USA). Non-parametric statistical tests were employed due to the non-normal distribution of the measured variables (Shapiro–Wilk Test; *p* ≤ 0.001). Descriptive analyses were conducted using frequency, percentage, range, mean, median, standard deviation (*SD*), rank, mean rank and interquartile range. Contingency tables and the χ^2^ (Chi-squared) test were used to analyse the distribution of participants by sex.

The totals of the needs identified by the participants in each area of the ENDE were calculated. The Mann–Whitney U test was used to analyse differences between two independent groups—by sex, age group, respondent type, and usual residence. To examine differences according to the level of ID, where there were three or more independent groups, the Kruskal–Wallis H test was used. To quantify the magnitude of the differences found between groups (effect size measures), Cliff’s Delta (δ) [29] was used for comparisons between two independent groups and Epsilon-squared (ε^2^) for comparisons involving three or more groups. A 5% error margin was assumed in the hypothesis testing, with a significance level of *p* ≤ 0.05.

## 3. Results

### 3.1. Descriptive Analyses of Perceived Needs

All but one of the participants identified needs in the areas of personal health (99.5%; *n* = 210), financial resources/money (82.9%; *n* = 175), health care and medical services (97.6%; *n* = 206), social services (93.8%; *n* = 198), physical and social barriers (95.7%; *n* = 202), and social relationships (91.5%; *n* = 193). In addition, 93.4% (*n* = 197) of the participants reported personal problems, 97.6% (*n* = 206) expressed concerns about the future, and all the participants identified solutions to these perceived needs.

Table 2 presents the descriptive statistics for the total number of needs identified by the participants in each of the areas in the ENDE interview, as well as the overall number of perceived needs.

Within each of these areas, the needs most frequently identified by participants were taking care of one’s health to stay well (49.8%; *n* = 105); maintaining the disability pension (67.3%; *n* = 142); help with technical aids or devices (71.1%; *n* = 150); help with understanding and completing important paperwork (70.6%; *n* = 149); help with healthcare issues (70.6%; *n* = 149); information about healthcare entitlements (47.9%; *n* = 101); and access to physiotherapy (31.3%; *n* = 66). With regard to personal problems, the most frequently reported issues were feelings of anxiety, stress or upset (46.9%; *n* = 99). Regarding the future, the most commonly expressed concerns were related to the desire for a good quality of life (74.4%; *n* = 157) and the fear of loneliness (41.2%; *n* = 87). Finally, the most common solutions mentioned were support with personal care (53.1%; *n* = 112), more and better residential facilities and sheltered housing (51.2%; *n* = 108), and more direct support staff (48.8%; *n* = 103).

### 3.2. Analysis of Differences According to Contrasting Variables

Statistically significant differences were found between men and women in the number of needs identified in the areas of health resources, physical and social barriers, as well as in the overall number of perceived needs. Differences between men and women were also found in the areas of personal problems, concerns about the future, and solutions. In all cases, men identified a greater number of needs than women (see Table 3 and Table 4).

Regarding differences based on usual place of residence (see Table 3 and Table 4), statistically significant differences were found in needs related to personal health, financial resources, and health care, as well as in the overall number of perceived needs. Compared to those living with family members, individuals living in care facilities identified fewer needs overall—signalling the areas of personal health, health care, and finances, in particular.

Regarding the two age groups, statistically significant differences were found between them in health care resources and solutions. Specifically, the younger participants identified more needs associated with health care resources, whereas the older participants identified a greater need for solutions.

The effect sizes can be interpreted as moderate [30], as the differences by place of usual residence and age group, while statistically significant, showed moderate relevance from a practical or clinical perspective [31].

With regard to differences by severity of the ID (see Table 4), statistically significant differences were found in the perceived needs related to social services, future outlook, and required solutions. Participants with moderate ID reported greater needs regarding social services, whereas those with severe ID identified more needs related to future outlook and solutions (see Table 3). The effect sizes found (ε^2^) suggest small or very small effects, except for future outlook, where moderate effect sizes were observed. This result indicates that although the differences were statistically significant, they would be of limited relevance from a practical or clinical perspective [31].

Finally, no statistically significant differences were found between the evaluation models; the number of perceived needs was similar irrespective of whether the individuals with ID responded to the interview alone or with the support of another person.

## 4. Discussion

The main objective of the study was to describe the perceived needs of a sample of individuals in the process of ageing with an ID. The results revealed that the participants with ID presented a broad, complex and multifaceted needs profile. The convergence of physical, mental and social limitations can give rise to a wide range of support needs across multiple areas [11], which aligns with the broad range of needs identified by our participants and evidenced by the results. Similarly, the high percentage of physical and social barriers identified by the participants in this study, along with their concerns about the future and personal problems, are consistent with findings in other studies that have highlighted the impact of isolation, social exclusion, and challenges to emotional well-being in older adults with ID, where emotional and interpersonal well-being emerges as one of the most pressing needs [32].

The fact that all the participants identified possible solutions is considered particularly relevant, as it indicates they showed both an interest in improving their situation and an ability to adapt to their circumstances. This result highlights the importance of encouraging strategies aimed at strengthening personal resources and self-efficacy among individuals with ID, as advocated by the Occupational Quality of Life Model [33].

Men identified a greater number of needs than women overall, and specifically in relation to health care resources and physical and social barriers. Differences were also found in personal problems, concerns about the future, and possible solutions. Our results are consistent with those reported by Alcedo et al. [23]; however, other studies have found no differences in perceived needs based on gender [15].

The differences found in our study may be explained by factors such as the possibility that men express their needs more openly, whereas women may tend to underestimate or downplay theirs for cultural reasons regarding care and dependency. These findings invite reflection on how gender influences the perception of one’s own needs in ageing people with ID. A more nuanced understanding of the perceived needs of individuals with ID is needed, using a person-centred approach that takes into account life course trajectories and includes gender-sensitive perspectives [33].

It was found that individuals with moderate ID perceived greater needs regarding social services, whereas those with severe ID identified more needs related to future outlook and possible solutions. These findings are consistent with research suggesting that the perceived needs of people with ID do not depend solely on environmental factors or access to services but also on aspects linked to the nature of the disability itself, such as levels of comprehension, awareness and symbolic representation of one’s own situation [11]. Individuals with moderate ID, who tend to present higher levels of adaptive functioning, may be more familiar with available community resources, which could explain why their demand for social services is greater. This profile is also consistent with studies that associate greater autonomy with increased awareness of social shortcomings, particularly in contexts where support is insufficient or inconsistent [32].

In contrast, the greater preponderance of perceived needs among individuals with severe ID regarding the future and potential solutions may be linked to a higher level of dependence on services and support figures, as well as to greater exposure to situations of structural vulnerability [34].

The results revealed statistically significant differences in perceived needs according to the individual’s usual place of residence. Specifically, those living in care homes identified fewer needs overall, specifically in the areas of personal health, healthcare services and financial resources, than those living with their families.

These findings are consistent with the literature that suggests that the residential context plays a key role in the identification and fulfilment of the needs of individuals with ID. Specialised residential care settings often provide more structured formal support, better access to healthcare services, professionalised direct care, and a degree of financial stability, which may explain the lower incidence of unmet needs perceived in these areas [10,35]. In contrast, individuals with ID that live in family settings may face greater difficulties in accessing healthcare or specialised services, particularly when the family network have to contend with financial constraints, lack the necessary knowledge about available resources, or struggle to manage complex age-related care needs [11,32]. Such situations are further exacerbated when the family carers themselves are also ageing, which, in turn, could lead to a breakdown in care or total collapse of the support system [10].

The participants aged 40 to 54 identified a greater number of needs related to access to health care resources, whereas those aged 55 to 75 expressed a greater demand for solutions. These findings are consistent with previous studies which showed a tendency for the younger cohort of ageing individuals to perceive more barriers in accessing health services, which may be due to a greater awareness of their own limitations or to the fact that they are moving towards the latter ageing period where they are expected to show greater autonomy in managing their own health care [11,23]. Older individuals, in contrast, tend to focus more on seeking practical solutions to improve their daily wellbeing when facing age-related challenges such as comorbidities and functional decline. This is reflected in a greater demand for care-related strategies and resources [36,37].

Longitudinal research on ageing with intellectual disabilities shows that although the decline in health tends to increase with age, older individuals often maintain reasonable levels of well-being and satisfaction—partly due to increasingly better resource management and adaptive coping skills [36]. This may explain why, despite perceiving fewer potential health care shortcomings, they express a greater need for solutions that would allow them to preserve their quality of life, particularly in cases where formal supports are inadequate.

No differences were found in perceived needs between the participants who completed the interview themselves and those who did so with the help of another person. These findings potentially support the idea that individuals with ID can provide valid and reliable assessments of their own needs, provided that adapted instruments and appropriate communication strategies are used. These results also underline the importance of consulting people with intellectual and developmental disorders about their needs and health care throughout the entire research process [38].

We consider that the results of the present study may strengthen the need to adopt an intensive personalised approach to service planning that takes into account the level of support required by the individual as well as their capacity to adapt and express themselves [39]. In this regard, tools which are sensitive to the individual’s level of intellectual disability—such as the ENDE interview [23]—make it possible to capture subtle differences in how individuals understand and express their needs.

However, this study also presented several limitations worth mentioning. Firstly, since a cross-sectional design was used, it was not possible to capture potential temporal dynamics in the analysed variables. Secondly, although the sample was large and representative, it was limited to two specific geographical areas, which may affect the generalisability of the findings to other regions with different care and social and health support models. Such differences in care models may complicate comparisons and limit the drawing of conclusions [40]. Finally, although the ENDE interview is a validated and appropriate tool for assessing needs in individuals with intellectual disabilities [23], the respondent’s degree of comprehension—even when supported by another person—may have affected how they perceived their needs, thus potentially impacting the reliability of some of the responses.

Conducting longitudinal studies that examine the evolution of perceived needs throughout the ageing process would make it possible to identify life trajectories and critical moments in the ageing process of people with ID. We believe that by expanding the study to include participants from different care and social and health support models, as well as incorporating advanced methodological strategies that ensure cognitive and communicative accessibility when collecting the data, the findings could be more easily generalised to the different support models and to people with ID with greater support needs.

There is international consensus that older adults with ID should enjoy the same rights as those without and therefore should have equal access to the same services and care as other adults [41]. It is essential that public administrations review current service models and adapt their social and health care programmes to respond to the growing number of ageing people with ID. Society needs policies that promote positive ageing for individuals with ID [42].

## 5. Conclusions

In conclusion, ageing adults with ID showed a broad and multifaceted profile of needs, with differences observed by sex, severity of ID, place of residence, and age group. The participants’ identification of possible solutions underscored, on the one hand, their adaptability to their circumstances and, on the other, the need to promote strategies that strengthen the personal resources and self-efficacy of ageing people with ID.

Understanding the perceived needs of people with ID who are in the process of ageing will facilitate the design of such policies and aid in the development of prevention and quality-of-life promotion programmes aimed at fostering self-determination. Promoting a long, healthy and dignified life for people with ID is not only a healthcare challenge but also an ethical, social, and legal responsibility.

## Figures and Tables

**Table 1:** Characteristics of the Participants.

		*n*	%	Contingency Table by Sex
Age group	42 to 55	111	53	*χ*^2^ = 0.089; *gl* = 1; *p* = 0.437
	56 to 75	110	47	
Level of Intellectual Disability	Mild	117	55.5	*χ*^2^ = 1.02; *gl* = 2; *p* = 0.600
	Moderate	29	13.7	
	Severe	65	30.8	
Recognised Level of Dependency	No recognised level	150	71.1	*χ*^2^ = 2.420; *gl* = 2; *p* = 0.298
	Level 1	38	18	
	Level 2	23	10.9	
Person who completed theENDE) interview	The individual with ID by themselves (self-application)	106	50.2	*χ*^2^ = 0.04; *gl* = 1; *p* = 0.842
	The individual with ID with a support person	105	49.8	
Usual residence (where and with whom)	With partner, children or other family members	123	58.3	*χ*^2^ = 4.795; *gl* = 1; *p* = 0.029
	In a care home	88	41.7	

Note: ENDE: Interview for the Needs Evaluation of Ageing People with Disabilities.

**Table 2:** Descriptive statistics of the number of identified needs. ENDE areas.

ENDE Areas	*M*	*SD*	*Mdn*	*Min*	*Max*	*Range*	*I-Q Range*
Personal health (PH)	2.25	1.08	2	0	5	4	2
Economic resources (ERs)	1.5	0.73	1	0	4	3	1
Health resources (HRs)	3.02	1.74	3	0	9	8	1
Social services resources (SSRs)	2.39	1.48	2	0	6	5	1
Physical and social barriers (PSBs)	2.46	1.40	2	0	6	5	2
Support measures and networks (SMNs)	1.95	1.09	2	0	6	5	1
Total perceived need (TPN)	12.81	5.94	11	2	34	32	9
Personal problems (PPs)	3.37	2.57	3	0	14	13	3
Future outlook (FO)	3.23	2.02	3	0	8	7	4
Solutions required (SRs)	10.12	8.24	7	1	31	30	11

Note: M: Mean; SD: Standard Deviation; Mdn: Median; Min: Minimum; Max: Maximum; I-Q Range: Inter-quartile Range.

**Table 3:** Descriptive statistic (Average Range) of the number of identified needs by sex, usual residence, severity of ID and age groups. ENDE areas.

ENDE Areas *	Sex		Usual Residence		Severity of ID			Age Group	
	Female	Male	Care Home	Family	Mild	Moderate	Severe	42–55	56–75
PH	98.88	113.99	89.88	117.54	99.04	123.69	110.64	103.35	108.95
ERs	99.96	112.61	95.71	113.36	102.24	118.55	107.18	105.91	106.10
HRs	94.44	119.68	93.62	114.86	105.13	120.71	101.01	97.99	114.89
SSRs	103.75	107.74	106.07	105.95	96	120.51	117.51	109.11	105.55
PSBs	95.45	118.39	98.98	111.02	104.19	126.83	99.97	106.88	105.02
SMNs	102.37	109.51	109.55	103.46	98.79	120.62	112.45	110.76	100.72
TPN	95.35	118.52	94.84	113.98	99.96	125.64	108.11	104.68	107.46
PPs	94.77	119.26	103.59	107.73	101.75	101.22	115.78	109.93	101.64
FO	95.75	118.01	106.07	105.95	91.09	108.31	131.82	110.21	101.33
SRs	97.79	115.39	111.06	102.38	94.7	117.03	121.42	114.07	97.04

Note: * see Table 1.

**Table 4:** Mean differences according to predictor variables.

ENDE Areas *	Sex		Usual Residence		Severity of ID		Age Group	
	*p*	δ	*p*	δ	*p*	ε^2^	*p*	δ
PH	0.06	0.14	<0.01	0.26	0.09	0.01	0.48	0.06
ERs	0.10	0.12	<0.05	0.16	0.36	0	0.98	0
HRs	<0.01	0.24	<0.05	0.20	0.32	0	<0.05	0.16
SSRs	0.62	0.04	0.98	0	<0.05	0.03	0.42	0.06
PSBs	<0.01	0.22	0.14	0.12	0.11	0.01	0.82	0.02
SMNs	0.37	0.06	0.45	0.06	0.11	0.01	0.21	0.10
TPN	<0.01	0.22	<0.05	0.18	0.12	0.01	0.74	0.02
PPs	<0.01	0.24	0.62	0.04	0.28	0	0.31	0.08
FO	<0.01	0.22	0.98	0	<0.01	0.08	0.28	0.08
SRs	<0.05	0.16	0.30	0.08	<0.01	0.03	<0.05	0.16

Note: * see Table 1; *p* = Associated probability; Effect size: δ = Cliff’s Delta; ε^2^ = Epsilon-squared.

## Data Availability

The raw data (de-identified data and analysis scripts) supporting the conclusions of this article will be made available by the authors on request.
